# Adaptive iron utilization compensates for the lack of an inducible uptake system in *Naegleria fowleri* and represents a potential target for therapeutic intervention

**DOI:** 10.1371/journal.pntd.0007759

**Published:** 2020-06-18

**Authors:** Dominik Arbon, Kateřina Ženíšková, Jan Mach, Maria Grechnikova, Ronald Malych, Pavel Talacko, Robert Sutak

**Affiliations:** 1 Department of Parasitology, Faculty of Science, BIOCEV, Charles University, Vestec, Czech Republic; 2 BIOCEV proteomics core facility, Faculty of Science, BIOCEV, Charles University, Vestec, Czech Republic; Georgetown University, UNITED STATES

## Abstract

*Naegleria fowleri* is a single-cell organism living in warm freshwater that can become a deadly human pathogen known as a brain-eating amoeba. The condition caused by *N*. *fowleri*, primary amoebic meningoencephalitis, is usually a fatal infection of the brain with rapid and severe onset. Iron is a common element on earth and a crucial cofactor for all living organisms. However, its bioavailable form can be scarce in certain niches, where it becomes a factor that limits growth. To obtain iron, many pathogens use different machineries to exploit an iron-withholding strategy that has evolved in mammals and is important to host-parasite interactions. The present study demonstrates the importance of iron in the biology of *N*. *fowleri* and explores the plausibility of exploiting iron as a potential target for therapeutic intervention. We used different biochemical and analytical methods to explore the effect of decreased iron availability on the cellular processes of the amoeba. We show that, under iron starvation, nonessential, iron-dependent, mostly cytosolic pathways in *N*. *fowleri* are downregulated, while the metal is utilized in the mitochondria to maintain vital respiratory processes. Surprisingly, *N*. *fowleri* fails to respond to acute shortages of iron by inducing the reductive iron uptake system that seems to be the main iron-obtaining strategy of the parasite. Our findings suggest that iron restriction may be used to slow the progression of infection, which may make the difference between life and death for patients.

## Author summary

*Naegleria fowleri* is a unicellular amoeba living in warm freshwater that is able to infect humans and cause a serious and mostly fatal disease with rapid progression. When water with the amoeba enters the nose, *Naegleria* penetrates the mucosa and invades the human brain, where it destroys cells and causes massive inflammation. It is a rare infection with unspecific symptoms, which slows the critical process of identifying the cause of the disease. Iron is a necessary element for all living organisms used in many biological pathways; therefore, iron acquisition and iron metabolism have the potential to be exploited against this parasite to clear or slow the infection. It was discovered that *N*. *fowleri* is unable to efficiently regulate iron uptake in an environment with a low iron concentration and merely changes its energy metabolism to handle the lack of this element. Because of this limited response, *N*. *fowleri* is more sensitive to low iron conditions than are human cells, and treatment by iron chelators has the potential to kill the pathogen or slow the infection in the host.

## Introduction

There are several free-living protists that, under certain conditions, are able to parasitize suitable hosts. The genus *Naegleria* consists of several species, the most studied of which are *Naegleria gruberi* and *Naegleria fowleri*, the latter being widely known as the “brain-eating amoeba”. Its rather vivid nickname is attributed to the condition known as primary amoebic meningoencephalitis (PAM). The amoeba is found in warm freshwaters across most continents and can transition between three distinguishable forms: a durable cyst, trophozoite amoeba and mobile flagellate [1]. Its presence in water is a health risk for people participating in recreational activities involving bodies of water [2]. The disease is acquired when *N*. *fowleri* trophozoites forcefully enter through the nasal cavity, invade the olfactory neuroepithelium and follow the olfactory nerve to the brain, which is their final destination [3]. The disease has a rapid onset, and the nonspecific symptoms resemble those of more common bacterial or viral meningoencephalitis–headache, fever, vomiting–with rapid progress, causing seizures, coma, and death [4,5]. Since PAM occurs commonly in healthy individuals, *N*. *fowleri* is regarded not as an opportunistic parasite but as a pathogen [3]. The fatality rate for PAM is reported to be above 97% [6]. Treating PAM is difficult because the symptoms are typical of other maladies and have rapid onset and because no specific or efficient medication is available for ameliorating the disease. For successful treatment, it is crucial that a correct diagnosis is made quickly and that therapy is immediately delivered. The currently accepted treatment includes administering several medications simultaneously, such as amphotericin B, fluconazole, rifampin, azithromycin and/or miltefosine, in combination with methods that decrease brain swelling [4,7]. Thus, the need for an efficient cure or at least novel compounds that will slow the progression of the infection persists. Among other free-living amoebae, that can cause life-threatening diseases are species of *Acanthamoeba*, *Balamuthia* and *Sappinia*. These amoebae cause rare granulomatous amoebic encephalitis, a deadly disease with symptoms similar to PAM but that progress more slowly and with additional manifestations [8,9]. Nevertheless, the issues with battling these diseases, such as difficulty making a diagnosis, are the same.

There is very limited knowledge about the biochemistry and physiology of *N*. *fowleri*, and practically nothing is known about its metabolism of iron, even though this metal may represent the parasite’s Achilles heel. Iron is an essential constituent of many biochemical processes, including redox reactions, the detoxification of oxygen, and cell respiration, and is a cofactor of various enzymes [10]. The ubiquitous role of iron is mainly due to its ability to transition between different oxidation states, enabling to participate in redox reactions, often in the form of iron-sulfur clusters [11]. Iron is essential for virtually all known forms of life, and its availability is shown to be the factor that limits organism growth in certain locations [12]. Its acquisition becomes especially challenging for pathogens that inhabit another living organism, as demonstrated by *Plasmodium*, in which iron-deficient human and mouse models manifest unfavorable conditions for parasite development; therefore, iron is a limiting factor for parasite propagation [13,14]. As a defense mechanism, mammals minimize the presence of free iron in their body using several proteins, such as lactoferrin, ferritin or transferrin, which bind the metal, decreasing its bioavailability [15]. Parasitic organisms are known to have adopted various means of acquiring iron from their environments, ranging from engaging in opportunistic xenosiderophore uptake to expressing specific receptors for mammalian iron-containing proteins [16]. This two-sided competition between pathogens and their hosts indicates the importance of iron metabolism in disease and underlines the importance of further research on this topic in the search for new methods of therapeutic intervention.

In this study, we demonstrate how *N*. *fowleri* acquires iron from its environment and how it adapts to iron-deficient conditions and we propose that the amoeba iron metabolism can be exploited to the advantage of PAM patients. We tested several potential iron uptake mechanisms and observed that the iron uptake system was not induced under iron starvation conditions and that the parasite used no alternative metabolic pathway to adjust for the resulting iron deficiency. Proteomic and metabolomic investigations showed that *N*. *fowleri* responded to low iron levels by maintaining iron-containing proteins in mitochondria, while the activity of nonvital, mostly cytosolic, iron-requiring pathways declined. These findings revealed a possible exploitable weakness in the survival strategy of the amoeba within the host. Although the host brain is relatively rich in iron content [17], not all of the iron is readily available for the parasite to use, as we have shown that it is not able to utilize iron bound to transferrin, the main source of iron in the human brain [18]. Thus, by using artificial chelators to decrease the availability of iron in this organ, we show a possible complementary antiparasite strategy, which is already utilized for different purposes, against this deadly pathogen [19].

## Results

### *N*. *fowleri* uses an uninducible reductive mechanism to acquire iron, while transferrin is not utilized

**Fig 1:**
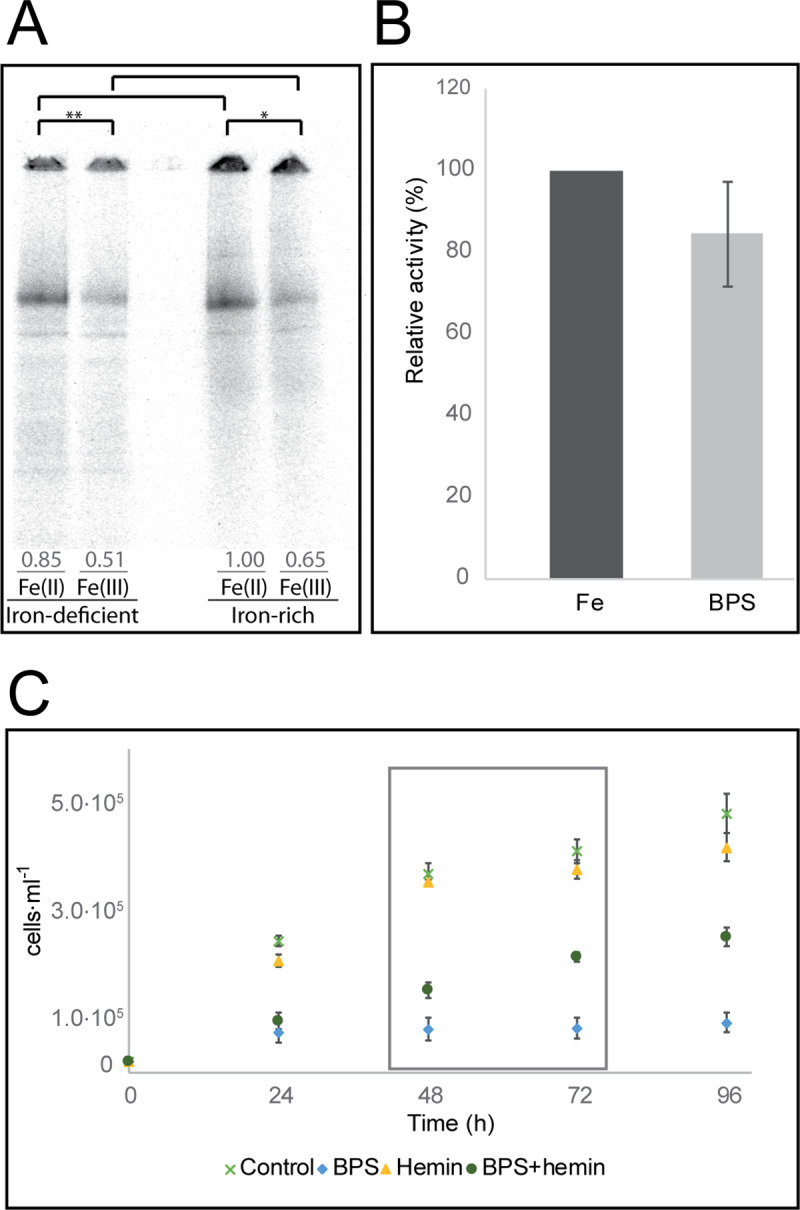
Iron uptake in *N*. *fowleri*. (A) Ferrous and ferric iron uptake by *N*. *fowleri* precultivated under iron-rich and iron-deficient conditions. Autoradiography of blue native electrophoresis gels of whole cell extracts from *N*. *fowleri* previously cultivated for 72 hours under iron-deficient conditions (25 μM BPS) or iron-rich conditions (25 μM Fe-NTA) and further incubated with ^55^Fe(II) (ferrous ascorbate) or ^55^Fe(III) (ferric citrate). Equal protein concentrations were loaded, and the loading control is shown in S1(A) Fig. The gel is representative of three independent replicates. Numbers indicate average relative densitometry values for the appropriate lines. Significant differences between band densitometry values are denoted by asterisk (*, *p*<0.05; **, *p*<0.01). (B) Ferric reductase activity under iron-rich and iron-deficient conditions. Relative values of *N*. *fowleri* ferric reductase activities in the iron-rich (Fe) and iron-deficient (BPS). The difference between the two conditions was not significant, p-value >0.05. Data are presented as the relative percentage ± SD from three independent replicates. (C) Growth restoration of *N*. *fowleri* in culture by hemin under iron-deficient conditions. Cells in regular growth medium (control) and with 50 μM hemin (hemin) exhibited similar levels of propagation, while the cells under iron-deficient conditions, achieved with 50 μM chelator bathophenanthroline disulfonic acid (BPS), showed stagnated growth in the first 24 hours. The addition of 50 μM hemin to the iron-deficient medium (BPS+hemin) partially restored culture growth. The boxed area indicates the time from which growth restoration was calculated. Data are presented as the means ± SD from four independent replicates.

Iron is generally available in the environment in two distinct oxidation states. Due to the different solubilities of the two forms, many organisms use ferric reductase to reduce ferric iron to ferrous iron, which is more soluble and therefore easier to assimilate. To explore the mechanism by which *N*. *fowleri* acquires iron from its surroundings, cell cultures were supplemented with different sources of iron: ^55^Fe-transferrin, ^55^Fe(III)-citrate and ^55^Fe(II)-ascorbate. For all the experiments conducted in this study, unless stated otherwise, iron deprivation was achieved using 25 μM of the common extracellular chelator bathophenanthroline disulfonic acid (BPS) to create a condition in which cell growth was significantly affected but microscopy showed no encystation or flagellated forms, and the cells did not lose their ability to multiply or attach to the surface. This condition was an important prerequisite, particularly for the proteomic and transcriptomic analysis, where very complex changes are accompanied by unfavorable cell processes that could bias the data. To achieve iron-rich conditions in this study, the cultivation medium was supplemented with 25 μM ferric nitrilotriacetate (Fe-NTA). The results presented in Fig 1A show that ferrous iron is taken up and incorporated into intracellular protein complexes more rapidly than was its trivalent counterpart. Densitometry of the radioactive signal in dried native electrophoresis gels was used to calculate the difference in iron utilization between the two forms: The utilization of ferric iron in comparison to ferrous iron was decreased in iron-rich cells to 65% (±8%; p-value <0.05) and in iron-deficient cells to 61% (±3%; p-value <0.01), suggesting the involvement of ferric reductase in the effective assimilation. Surprisingly, there was no significant difference in iron uptake between cells preincubated in the iron-rich and iron-deficient conditions, showing that the organism failed to stimulate its iron uptake machinery. The insignificant uptake of transferrin, shown in S1(B) Fig, means that this protein is not a viable source of iron for *N*. *fowleri*, corresponding to the fact that it is not found in the usual water habitat of this organism.

To confirm that *N*. *fowleri* uses a reductive iron uptake mechanism, the cell cultures were treated with a ferric iron radionuclide in the presence and absence of the ferrous iron chelator BPS. The results shown in S1(C) Fig demonstrate that, while *N*. *fowleri* readily incorporates ferric iron into its protein complexes, BPS chelates the initially reduced ferrous iron and prevents it from being further utilized, confirming that ferric iron uptake requires a reduction step.

We have shown that neither ferrous nor ferric iron uptake is induced by iron starvation (Fig 1A). Considering the involvement of the reduction step needed for *N*. *fowleri* iron acquisition, the activity of a ferric reductase was assessed in cells preincubated under iron-rich and iron-deficient conditions. As shown in Fig 1B, measurements of ferric reductase activity revealed that the level of ferric iron converted to ferrous iron was not significantly changed in the iron-deprived cells (p-value >0.05). This finding confirms that *N*. *fowleri* cannot efficiently adjust the rate of iron acquisition from its surroundings to overcome its dependence on iron availability.

Heme-containing proteins are potential source of iron or heme for several parasitic protists [20,21]. Although we did not identify a homolog of heme oxygenase in the *N*. *fowleri* genome, which is necessary for the breakdown of heme and the release of iron, we tested the effect of the presence of hemin in the cultivation medium on the growth of the amoebae. As shown in Fig 1C, cells cultivated in regular growth medium had a propagation pattern similar to that of the cells grown in medium supplemented with 50 μM hemin; therefore, at the given concentration, hemin was not toxic to *N*. *fowleri*, nor did it significantly support its growth under standard cultivation conditions. The profound iron-deficient conditions achieved with 50 μM BPS arrested culture growth to 31% (±12%) during the first 24 hours, and the propagation at later time points remained below this value. The addition of 50 μM hemin partially restored culture growth to 42% (±10%; p-value <0.01) after 48 hours, and by 72 hours, the growth reached 52% (±7%; p-value <0.01). Therefore, it appears that the hemin compound is used as a partial source of iron for the pathogen, although it cannot fully support its growth when other sources of iron are limited.

### Proteomic analysis findings illuminate the iron-starvation response of *N*. *fowleri*, while the transcriptomic analysis does not reflect the proteomic changes

Since iron has an irrefutable role as a cofactor for various enzymes and its metabolism is dependent on a great number of proteins, we aimed to examine the overall effect of iron availability on the *N*. *fowleri* proteome. Therefore, we compared the whole-cell proteomes of cells grown under iron-rich and iron-deficient conditions, and we have additionally analyzed membrane-enriched fractions of cells to obtain a higher coverage of the membrane proteins. The aim was to reveal the metabolic remodeling accompanying iron starvation and to identify proteins responsible for iron homeostasis, such as membrane transporters, signaling and storage proteins or proteins involved in iron metabolism, for example, the formation of iron-sulfur clusters.

**Fig 2:**
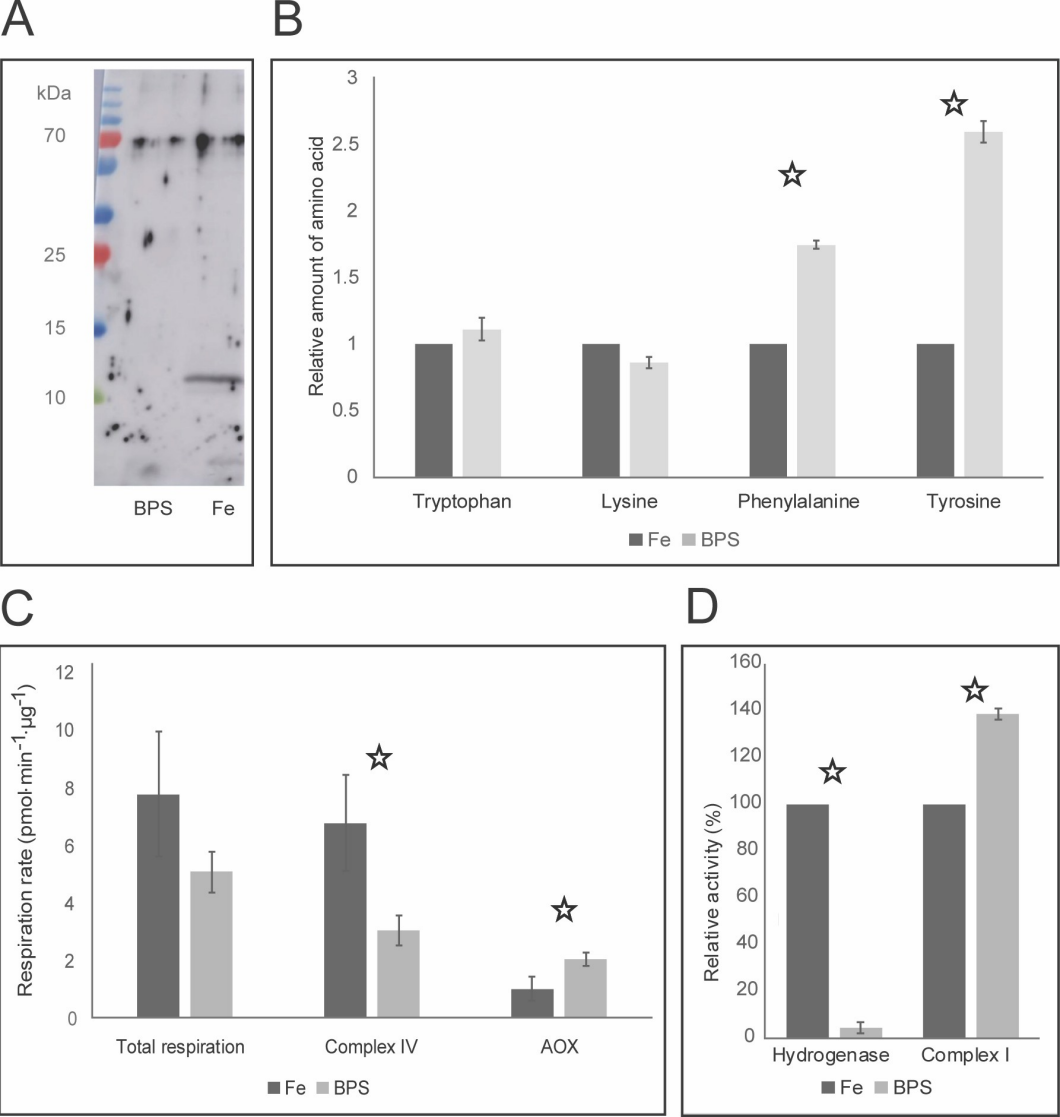
Effect of iron deficiency on *N*. *fowleri*. (A) Downregulation of *N*. *fowleri* hemerythrin under iron-deficient conditions. Results from the Western blot analysis of cells cultivated under iron-rich (Fe) and iron-deficient (BPS) conditions using an antibody against *Naegleria gruberi* hemerythrin. Equal protein concentrations were loaded, and the loading control is shown in S1(D) Fig. The gel represents one of three independent replicates. (B) Cellular content of selected amino acids in *N*. *fowleri* cells cultivated under iron-rich and iron-deficient conditions. Relative amounts of phenylalanine and tyrosine were significantly increased under the iron-deficient condition, while those of tryptophan and lysine remained unchanged. t-test p-values <0.01 are marked with a star. The total protein concentration was equal in all the samples, and the values shown are relative to those under the iron-rich conditions for each amino acid. Fe, cells cultivated under iron-rich conditions; BPS, cells cultivated under iron-deficient conditions. Data are presented as the means ± SD from three independent replicates. (C) Respiration of *N*. *fowleri* grown under iron-rich and iron-deficient conditions. Using selective inhibitors of complex IV (potassium cyanide) and of alternative oxidase (salicyl-hydroxamic acid), the contribution of alternative oxidase and complex IV activity was assessed with respect to total respiration levels. AOX, alternative oxidase; Fe, cells cultivated under iron-rich conditions; and BPS, cells cultivated under iron-deficient conditions. The t-test p-values <0.01 are marked with a star. Data are presented as the means ± SD from five independent replicates. (D) Activity of hydrogenase and NADH:ubiquinone dehydrogenase (complex I) under iron-rich and iron-deficient conditions. While hydrogenase was significantly downregulated, complex I was significantly upregulated as a result of the iron-deficient conditions. Relative values are shown. Fe, cells cultivated under iron-rich conditions; and BPS, cells cultivated under iron-deficient conditions. The t-test p-values <0.01 are marked with a star. Data are presented as the means ± SD from four independent replicates.

S1 Table lists the proteins that were significantly upregulated or downregulated under iron-deficient conditions based on the analysis of the *N*. *fowleri* whole-cell proteome, and S2 Table contains the regulated proteins in the membrane-enriched fraction. No major changes in encystation or flagellation markers or apoptotic pathway proteins between the two conditions were detected in the proteomic analysis. The proteins most relevant for this study are summarized in Table 1. The list of downregulated proteins based on whole-cell proteomics under iron-deficient conditions contained 20% of predicted iron-containing proteins, most of which were nonheme enzymes such as desaturases and oxygenases, or hydrogenase (NF0008540) and its maturases HydE (NF0081220) and HydG (NF0081230). Importantly, most of the downregulated iron-containing proteins were typically located outside mitochondria. The dramatic downregulation of the hemerythrin homolog (NF0127030) was confirmed by Western blotting using an antibody generated in-house against *N*. *gruberi* hemerythrin (Fig 2A).

**Table 1:** Effect of iron deprivation on the abundance of selected *N*. *fowleri* proteins.

Gene ID	Protein	Whole cell (membrane fraction) proteomics fold-change in iron deficiency	*p*-value
NF0117840	Protoglobin	ND in iron-deficient	NA
NF0127030	Hemerythrin	-7045.1	<0.05
NF0119700	Hemerythrin	-11.4	NA
NF0106930	Phenylalanine hydroxylase	-5.7	<0.05
NF0008540	Hydrogenase	-3.7	<0.05
NF0081220	HydE	-2.8	<0.05
NF0081230	HydG	-2.4	<0.05
NF0036800	Iron-containing cholesterol desaturase daf-36-like	-2.0 (-3.4)	NA (<0.05)
NF0001470	Tyrosine aminotransferase	-2.0	<0.05
NF0073220	Homogentisate 1,2-dioxygenase	-1.8	<0.05
NF0084900	4-Hydroxyphenylpyruvate dioxygenase	-1.5	<0.05
NF0121200	Catalase	-1.3	<0.05
NF0044680	Iron III Superoxide Dismutase	1.1	0.47
NF0004720	Alternative oxidase	1.5	<0.05
NF0014630	Mitochondrial carnitine/acylcarnitine transferase *	ND (1.5)	NA (0.39)
NF0015750	Manganese superoxide dismutase	1.6	<0.05
NF0079420	Mitoferrin *	1.8 (1.7)	<0.05 (0.08)
NF0020800	Fe superoxide dismutase	1.8	<0.05
NF0048730	Glutathione peroxidase	2.0	<0.05
NF0071710	Deferrochelatase	2.3	<0.05
NF0060430	IscU *	3.0	<0.05
NF0001440	Cysteine desulfurase	3.7	<0.05
NF0030360	Cu-Zn superoxide dismutase	5.8	<0.05
NF0001420	Mitochondrial phosphate carrier protein *	8.0 (8.4)	NA (<0.05)

Proteins were manually annotated using HHPRED or by sequence alignment with homologous proteins from other organisms (denoted with a star). The fold change based on proteomic analysis of whole cells and membrane fractions (for selected proteins; in brackets) is given for cells cultivated under iron-deficient conditions. Significantly regulated proteins (with a fold change above 2.3 and *p*-value not higher than 0.05) are highlighted in green. ND, not detected; NA, not applicable.

In the phenylalanine catabolism pathway, all three iron-dependent enzymes, phenylalanine hydroxylase (NF0106930), p-hydroxyphenylpyruvate dioxygenase (NF0084900) and homogentisate 1,2-dioxygenase (NF0073220), were downregulated in iron-deprived cells, even though the decrease in the expression of the last two enzymes was below our set threshold.

The proteins upregulated under iron-deficient conditions included two essential mitochondrial components of iron-sulfur cluster assembly machinery, namely, cysteine desulfurase (NF0001440) and iron-sulfur cluster assembly enzyme IscU (NF0060430); two mitochondrial transporters, phosphate carrier (NF0001420) and iron-transporting mitoferrin (NF0079420); and a potential homologue of deferrochelatase (NF0071710). The increase in the expression of mitoferrin was below our set threshold, but the results from the comparative analysis of the membrane-enriched fraction of *N*. *fowleri* grown under different iron conditions (S2 Table and Table 1) confirmed the upregulation of this transporter as it did for the mitochondrial phosphate carrier. Additionally, in the membrane-enriched proteomic analysis, a carnitine/acylcarnitine transferase (NF0014630) was identified and found to be slightly, although not significantly, upregulated.

Iron metabolism is interconnected with the production and detoxification of reactive oxygen species. Among the antioxidant defense proteins, one family of superoxide dismutases contains iron as a cofactor. Our proteomic analysis showed that while iron-dependent SODs (NF0020800 and NF0044680) were not significantly changed, Cu-Zn-dependent SOD (NF0030360) was upregulated under iron-deficient conditions, suggesting a compensatory mechanism for the mismetallated iron-dependent enzyme. Other radical oxygen species detoxification enzymes, such as catalase (NF0121200), manganese SOD (NF0015750), or glutathione peroxidase (NF0048730), were not significantly regulated under iron-deficient conditions.

To uncover a broader spectrum of the affected proteins that we were unable to detect in the proteomic analysis, we performed a transcriptomic analysis of *N*. *fowleri* grown under the same conditions of iron availability as used in the proteomic analysis (S3 Table). To our surprise, the data did not correspond to the proteomics results. Overall, the number of genes that were significantly changed in the transcriptomic analysis results was 287 (182 upregulated and 105 downregulated genes), which is more than those regulated in whole cell proteomic analysis; however, the only genes regulated in the same way as the proteins observed in the proteomic analysis were hemerythrins, protoglobin (NF0117840) and the iron-containing cholesterol desaturase daf-36-like (NF0036800). The response of *N*. *fowleri* to iron starvation is thus most likely posttranslational. The large proportion of nonheme iron-containing enzymes among the downregulated proteins indicates that the degradation of mismetallated/misfolded/nonfunctional proteins plays an important role in iron-induced proteome changes. To confirm the physiological relevance of the changes observed at the proteome level, we further investigated selected biochemical pathways that were indicated by the proteomic results.

### *N*. *fowleri* responds to iron deficiency by metabolic rearrangement that favors mitochondria

Considering the iron-dependent changes in the abundance of proteins participating in the phenylalanine degradation pathway, the next aim of this study was to analyze this effect by directly detecting metabolites, namely quantifying the cellular levels of corresponding amino acids by metabolomics. Cells grown under iron-rich and iron-deficient conditions were lysed, the protein concentrations were equalized, and the resulting material was analyzed for amino acid content by liquid chromatography coupled with mass spectrometry. Since the phenylalanine degradation pathway was predicted to be downregulated by the proteomic analysis results, we assessed the intracellular amounts of phenylalanine and tyrosine, amino acids likely affected by a decrease in iron-dependent enzymes. As expected, under iron-deficient conditions, the amount of phenylalanine was **s**ignificantly increased, by 75% (±3%; p-value <0.01), and tyrosine was increased by 160% (±8%; p-value <0.01) (data are shown in Fig 2B). As controls, relative amounts of tryptophan and lysine, the levels of which were not expected to change according to iron levels, were determined in the same samples.

*N*. *fowleri* cells possess lactate dehydrogenase and are therefore potentially able to produce lactate from pyruvate while replenishing the level of NAD^+^ cofactor, thereby utilizing cytosolic pathways for energy metabolism. To analyze the effect of iron starvation on this metabolic pathway, lactate production was analyzed by gas chromatography coupled with mass spectrometry detection. The level of intracellular lactate in the iron-deficient cells was decreased by 32% (±13%; p-value <0.05), showing that this metabolic pathway was not used in a compensatory strategy during iron deficiency.

Alternative oxidase (AOX), present in *N*. *fowleri*, is a part of the mitochondrial respiratory chain. It accepts electrons from ubiquinol to reduce the final electron acceptor, oxygen. Therefore, the function of AOX is similar to that of complex IV; however, the branching electron flow towards AOX bypasses some of the proton pumping complexes, decreasing the effect of the respiration chain. Using selective inhibitors of respiration complex IV and AOX enables the study of their participation in respiration. Here, the effect of decreased iron availability on the respiratory chain was determined. The results are shown in Fig 2C and demonstrate that the total respiration of the cells grown under iron-deficient conditions was decreased by 35%, although this change was not statistically significant (p-value >0.01), and that this decrease was based on the diminished activity of complex IV (55% decrease; p-value <0.01). In contrast, in the iron-deficient cells, the activity of AOX increased by 104% (p-value <0.01). This finding shows that AOX, despite being an iron-containing enzyme, is able to rescue respiration when iron deficiency causes a decrease in complex IV activity.

To further support the hypothesis that iron-starved *N*. *fowleri* maintains essential mitochondrial iron-dependent proteins at the expense of nonessential cytosolic proteins, we assessed iron-induced changes in the activity levels of mitochondrial NADH:ubiquinone dehydrogenase (complex I) and hydrogenase, which was shown to be cytosolic in *Naegleria* [22]. As shown in Fig 2D, the activity of hydrogenase was dramatically decreased, by 95%, under iron-deficient conditions (p-value <0.01), indicating that it is a dispensable component of the pathway when iron is scarce. In contrast, the activity level of mitochondrial complex I was increased by 39% under iron-deficient conditions (p-value <0.01), thus it was maintained as part of a vital pathway. Hence, the increased activity levels of the mitochondrial iron-containing enzymes AOX and complex I were able to rescue total mitochondrial respiration despite the decrease in the activity of complex IV. This finding demonstrates that the mitochondrial respiration chain is essential and maintained under iron-deficient conditions even if it is strongly iron demanding.

### Iron chelators significantly hinder *N*. *fowleri* growth

Iron plays a vital role in many biochemical processes; therefore, it is rational to expect that decreasing the bioavailability of iron in the surrounding environment would hinder cell growth. To determine the effect of iron on the propagation of *N*. *fowleri* in culture, three different iron chelators were added to the growth medium: bathophenanthroline disulfonic acid (BPS), 2,2′-dipyridyl (DIP) and deferoxamine (DFO). The compounds inhibited the growth of the cultures to different extents compared to the growth under iron-rich conditions (Table 2 and S2(A) Fig). The most potent effect in hindering culture growth was observed with the siderophore DFO. Both BPS and DIP had notably higher IC_50_ values.

**Table 2:** Iron chelator IC_50_ values for *N*. *fowleri* cultures after 48 hours.

Compound	IC_50_ (μM)
DIP	30.39 (±3.49)
BPS	17.01 (±2.42)
DFO	6.32 (±0.85)

DIP, 2,2′-dipyridyl; BPS, bathophenanthroline disulfonic acid; and DFO, deferoxamine. The data used for the IC_50_ extrapolation are depicted in S2(B) Fig.

Trophozoites of the amoebae feed on bacteria in their natural environment, making the bacteria potential sources of iron. Moreover, we presume that phagocytosis of human cells can represent one of *N*. *fowleri* virulence factors [23]. To investigate overall cell viability and to test whether *N*. *fowleri* cells induce phagocytosis as a potential way to acquire iron, the ability of iron-deficient cells to phagocytose bacteria was investigated. Quantification of phagocytosis was determined by flow cytometry using *Escherichia coli* that present increased fluorescence within acidic endocytic compartments. The values were calculated as percentages of amoebae in the total population with phagocytosed bacteria. Under iron-rich conditions, the percentage of phagocytosing amoebae was 70% (±6%), while under iron-deficient conditions, it was 53% (±4%). This significant decrease (p-value <0.01) shows that iron availability plays a role in this process. A representative dot plot from the flow cytometry data is shown in S3 Fig, including the controls of *N*. *fowleri* cells with no bacteria and a bacterial culture. A typical cell phagocytizing fluorescent bacteria is shown in the S1 video.

To show the extent to which iron-deficient *N*. *fowleri* is able to restore its own growth by phagocytosing bacteria, we presented attenuated *Enterobacter aerogenes* to amoebae cultivated under different iron conditions. As shown in S4 Fig, while the iron-deficient cells proliferated significantly more slowly than the iron-rich cells did, the addition of bacteria did not increase the proliferation of the cells cultivated under iron-deficient or iron-rich conditions. In contrast, the addition of iron to previously iron-deficient cultures fully reestabilished the original cell proliferation rate. In summary, iron is a vital element for *N*. *fowleri* cell propagation and chelators have a cytostatic effect on amoebae. Phagocytosis of bacteria is not a sufficient strategy of iron acquisition and is even suppressed during iron deficiency.

## Discussion

To maintain an optimal level of cellular iron in a hostile environment, such as host tissues, pathogens possess selective and effective mechanisms for iron uptake. These strategies of obtaining iron include the utilization of various sources from the host, including transferrin, lactoferrin or heme, and some parasites even exploit bacterial siderophores as sources of iron [16]. Transferrin, an abundant human blood protein that transports iron to various tissues, including the brain [18], serves as a viable source of iron for different parasites [16]. It was demonstrated that *N*. *fowleri* possesses proteases able to degrade human holotransferrin, although the study did not identify the intracellular fate of the iron [24]. Our study shows that *N*. *fowleri* does not appear to have the means of efficiently utilizing iron from this host protein (S1B Fig), perhaps because it is a facultative pathogen with no advantage of such an iron uptake mechanism in its natural environment. We further demonstrated the preference of *N*. *fowleri* for ferrous iron compared to ferric iron (Fig 1A), the inhibitory effect of ferrous iron chelator on ferric iron uptake (S1C Fig) and the presence of extracellular ferric reductase activity (Fig 1B). Based on these observations, we argue that the main strategy of iron acquisition by this parasite could be the reductive two-step iron uptake mechanism, as described for *Saccharomyces cerevisiae* [16].

*S*. *cerevisiae* possesses the ability to upregulate the expression of ferric reductase, which is responsible for the first step of the reductive iron uptake mechanism, up to 55 times under iron-deficient conditions [25], therefore increasing the rate of iron uptake. Our study shows that in *N*. *fowleri*, the activity of ferric reductase is not induced by iron starvation nor is the ferric and ferrous iron uptake and further incorporation of iron into cellular proteins (Fig 1A and 1B). The lack of an inducible iron uptake system has also been described in the parasite *Tritrichomonas foetus* [26]. However, contrary to *N*. *fowleri*, the obligatory parasite *T*. *foetus* appears to be able to utilize a wide range of iron sources, including host transferrin or bacterial siderophores. Another potential source of iron for *N*. *fowleri* can be heme from heme-containing proteins. The ability to utilize exogenous metal-containing porphyrins may be advantageous for protists feeding on bacteria. Moreover, *Naegleria* species phagocyte erythrocytes [27,28], and their ability to degrade hemoglobin using proteases was also described [24]. However, it appears that heme oxygenase is not present in the genome of *N*. *fowleri*; therefore, it is unlikely to be able to employ this enzyme to obtain iron from hemoglobin, as is the case of *T*. *foetus* [26]. A potential homologue of bacterial deferrochelatase, a protein able to directly acquire iron from heme [29], was identified as significantly upregulated under iron-deficient conditions by proteomic analysis (Table 1); however, further investigation is required to clarify the function of this protein. Nevertheless, hemin was shown to partially restore *N*. *fowleri* growth in very strong iron-deficient conditions (Fig 1C). Since *N*. *fowleri* requires exogenous porphyrins for growth [30], the fact that the addition of hemin partly suppresses the conditions of iron starvation can be attributed to a metabolic rearrangement towards heme-dependent pathways. Although phagocytosis could be an alternative strategy for obtaining iron in natural and host environments, according to our data, it does not appear to be utilized under iron-deficient conditions. We showed that the actual rate of phagocytosis was decreased in this case and that the extent of iron-deficient culture propagation could be restored by the addition of iron but not by attenuated bacteria (S4 Fig). Considering these findings, it is important to note that the relationship between decreased phagocytosis and decreased hemerythrin expression was previously described [31]. While *N*. *fowleri* is unable to utilize iron from transferrin, it is possible that phagocytosis is one of the strategies for obtaining iron from the host and thus may represent a virulence factor that can be diminished by iron starvation. This presents an opportunity to use chelation-based therapy to decrease the ability of the pathogen to gain access to the available iron from host tissues and thereby further increases the iron deficiency of the parasite.

Proteomic analysis proved to be a valuable resource in determining the cellular changes in *N*. *fowleri* induced by iron starvation and provided a foundation for further discoveries that are shown in this study (Table 1). The most fundamental finding of comparative proteomic analysis is that mainly cytosolic iron-containing proteins were downregulated when iron was limited. These proteins are likely components of nonessential pathways, such as the phenylalanine degradation, hydrogenase maturation and hydrogen production pathways. The accumulation of phenylalanine was observed in *S*. *cerevisiae*, where iron-deficient cells contained about 50% more of the amino acid than iron-rich cells [32]. On the other hand, mitochondrial iron-dependent proteins were generally unchanged, while some components of the iron-sulfur cluster synthesis machinery were upregulated under iron-deficient conditions, emphasizing the essential role of iron-dependent respiration process. This mitochondrial sequestration of iron to ensure respiration was confirmed by increased complex I activity with a concomitant decrease in the activity level of hydrogenase (Fig 2D) as well as a reduction in the iron-dependent catabolism of amino acids (Fig 2B). Consistent with this observation, the mitochondrial iron transporter was upregulated under iron-deficient conditions. Moreover, the carnitine/acylcarnitine carrier was identified in the membrane-enriched proteomic analysis, and its expression was slightly increased in iron-starved cells. This mitochondrial membrane-bound protein is involved in lipid metabolism, which was recently shown to be vital for *N*. *gruberi* [33]. Another mitochondrial transport protein, a phosphate carrier, was strongly upregulated under iron-deficient conditions, likely to compensate for impaired respiration and decreased ATP production in the mitochondria of iron-deficient cells.

*N*. *fowleri*, as well as *N*. *gruberi*, possesses an AOX in the mitochondria, and our study indicates one of the possible advantages of this respiratory chain element for these organisms. Under iron-deficient conditions, the activity of AOX was significantly increased (Fig 2C), even though it requires iron, suggesting that this unusual branch of the respiratory chain may take over a portion of the activity in the iron-demanding conventional pathway of respiratory complexes III, IV and cytochrome c, an observation noted in the nonpathogenic amoeba *N*. *gruberi* previously [34]. This could represent a favorable compensation pathway, even though the overall generation of the proton gradient and therefore ATP synthesis is hindered. In addition, we observed an increase in the activity of complex I (Fig 2D), supporting the claim that the respiration is shifted towards the less-efficient but also less iron-dependent AOX pathway. Considering the reduced efficiency of respiration by iron-starved cells and the presence of lactate dehydrogenase in the genome of *N*. *fowleri*, it would be reasonable to expect the employment of the lactate dehydrogenase pathway in the regeneration of the cofactor NAD^+^. Such an effect was observed in *Trichomonas vaginalis*, where the cells modulate this pathway as a way of compensating for the metronidazole-induced loss of hydrogenosomal metabolism [35]. However, our metabolomic analysis showed the opposite change; the production of lactate was decreased upon iron starvation, suggesting another, most likely nonessential, function of this pathway that is attenuated due to the hindered rate of overall energy metabolism. Another possible compensatory pathway, ethanol production, is improbable because of the absence of pyruvate decarboxylase or bifunctional aldehyde/alcohol dehydrogenase in the *N*. *fowleri* genome. The function of cytosolic hydrogenase, which was significantly downregulated in the iron-limited conditions together with its maturation factors, is unknown in *Naegleria*.

Consistent with our previous study of iron metabolism in *N*. *gruberi* [34], hemerythrin was dramatically downregulated under iron-deficient conditions (Fig 2A), while it appears to be an abundant protein under standard conditions, based on the intensities obtained in proteomic analysis from iron-rich cells. The involvement of hemerythrin in the iron metabolism of *Naegleria* is suggestive but unclear. The presence of unbound metals in the cell must be strictly regulated since the imbalance in iron homeostasis can lead to the formation of ROS, mismetallation or other anomalies leading to the incorrect function of proteins. The relationship between hemerythrin and defense against oxidative stress was previously suggested in bacteria [36,37] and so was the role of hemerythrin-related proteins in iron homeostasis [38] or oxygen sensing [39]. One of the basic mechanisms of maintaining the proper intracellular level of metals is the regulation of their acquisition. Since our study shows the lack of such regulation for iron, it is possible that the sequestration of toxic free iron, as well as its storage for use under iron-deficient conditions, is ensured by hemerythrin functioning as a cytosolic iron pool. This hypothesis is supported by the fact that hemerythrin is a nonheme, noniron-sulfur metalloenzyme and is among the most strongly regulated proteins by iron availability, and unlike other proteins, its regulation was detected even at the mRNA level. Another oxygen-binding protein with unclear function, protoglobin, was detected only under iron-sufficient conditions. The role of protoglobin in the metabolism of *N*. *fowleri* remains to be elucidated.

Due to the irreplaceable role of iron in cellular processes, it is unsurprising that iron chelators have the ability to hinder the proliferation of *N*. *fowleri* [40]. The different chelators used in this study have distinct properties that influence their impact on cells. DIP is a membrane-permeable compound with affinity to ferrous iron [41] and a ratio of three chelator molecules binding one molecule of metal [42]. BPS is a membrane-impermeable chelator that binds ferrous iron with a ratio of three chelator molecules to one metal ion [43]. Finally, DFO is a membrane-impermeable, ferric iron-binding siderophore [44] with a binding ratio of one iron per molecule [45]. Quite surprisingly, the only tested membrane-permeable iron chelator, DIP, had the highest IC_50_ value (30.39 μM; SD = 3.49). In comparison with BPS, with which it shares an affinity to ferrous iron and the same denticity, DIP was almost half as effective in inhibiting culture growth. BPS and DFO differed not only by the oxidation state of bound iron but also by the ratio of molecules bound to the chelated iron. Their IC_50_ values were 17.01 μM (SD = 2.42) and 6.32 μM (SD = 0.85), respectively, showing an apparent tendency of the approximately three-fold amount of BPS required to have the same effect as DFO, probably because of the binding ratio. Therefore, surprisingly, it appears that from the selected chelators, the membrane-impermeable chelators are more effective against *N*. *fowleri*. It is important to consider that the growth medium used in this study was adjusted to the amoeba requirements, while in its host, the parasite is expected to meet much harsher conditions. The inhibiting concentrations of chelators shown in this study were of a magnitude that can be achieved in the human body, such as has been shown for DFO [46], demonstrating that chelation therapy could be considered in the case of PAM. It must be emphasized, that experiments in this study were performed in axenic cultures under optimal growth conditions. Thus, the anticipated *in vivo* antiparasitic effect of suitable chelators may be more pronounced. This laboratory setting undoubtably differs from the natural habitat of the pathogen or the host, where the conditions are harsher, and many interactions take place; therefore, further *in vivo* experiments are required to extend these results towards practical utilization. Our study could not conclusively assess the viability of chelators as suitable therapeutics. However, based on our data, we believe that iron chelation therapy is a promising approach to hinder and attenuate the progress of the disease while not being hazardous to the host, since even long-term intensive exposure of iron chelators can be safely applied to humans [47]. Considering the severity of the disease, we do not assume that chelators may fully cure PAM in patients, but we are aiming to safely hinder the rapid progression of the infection, thus securing more time for correct diagnosis determination and to deploy effective combination therapy.

**Fig 3:**
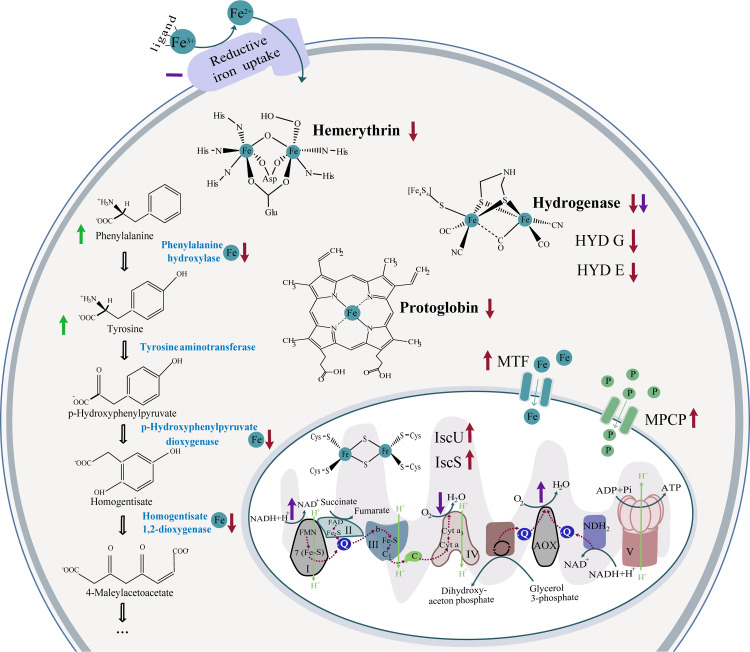
Illustration of the main effects of iron-deficient conditions on the selected cellular processes of *N*. *fowleri*. The results of proteomic analysis for selected proteins are depicted in red, the results from measured metabolite levels are in green and the assessed enzyme activities are in purple. Upwards pointing arrows stand for increased in the iron-deficient condition, downwards pointing arrows represent decreased in the iron-deficient conditions and dashes mean no significant change in the different iron conditions. Respiration chain complexes are represented by appropriate numbers, Fe represents iron-containing/involving protein/process. C, cytochrome C; MTF, mitoferrin; MPCP, mitochondrial phosphate carrier protein; P, phosphate; Q, ubiquinol/ubiquinone.

In conclusion, our study suggests that *N*. *fowleri* possesses only limited capabilities of adaptation to an iron-deficient environment and is surprisingly not able to utilize transferrin as an alternative source of the metal; neither can it effectively induce the rate of iron acquisition under iron starvation, reflecting the lifestyle of a facultative parasite with limited ability of survival in a host. The main strategy of acquiring iron appears to be reductive iron uptake. Proteomic analysis of the response to iron starvation demonstrated that a large amount of proteins downregulated under the iron-deficient conditions were nonmitochondrial and nonheme enzymes. The exception is the heme-containing protein protoglobin, whose expression is regulated at the transcriptional level, unlike the expression of most other affected proteins. Therefore, it can be hypothesized that the fundamental effect of iron deprivation is the degradation of mismetallated cytosolic proteins with a simultaneous increase in iron delivery to mitochondria and induction of iron-sulfur cluster synthesis machinery to ensure essential cell processes. The overall changes in cellular processes in the iron-deficient conditions discussed in this paper are illustrated in Fig 3. These findings are in agreement with our previous study focused on iron metabolism in the nonpathogenic model organism *N*. *gruberi* [34], where the mitochondrion was shown to be the center of the iron economy. Together, these results show that iron-deficiency is a highly unfavorable condition for *N*. *fowleri*, and targeted interference with its uptake could be an effective method of controlling the propagation or viability of this organism in the host.

## Materials and methods

### Statistical analysis

Unless stated otherwise, two-tailed Student’s *t*-test with two-sample equal variance was used to determine the *p*-values. For comparative proteomic analysis, the data was analyzed using the Perseus [48] package Maxquant [49] with two-tailed T-test of equal variance, S0 = 0.5, FDR = 0.01, quantification and normalization procedure MaxLFQ [50] was used.

Unless stated otherwise, all experiments were performed in three or more independent replicates, meaning parallel experiments on independently cultivated cultures.

### Organisms

*Naegleria fowleri*, strain HB-1, kindly provided by Dr. Hana Pecková (Institute of Parasitology, Biology Center CAS) was maintained in 2% Bacto-Casitone (Difco, USA) supplemented with 10% heat-inactivated fetal bovine serum (Thermo Fisher Scientific, USA), penicillin (100 U/ml) and streptomycin (100 μg/ml) in 25 cm^2^ aerobic cultivation flasks at 37°C. When required, the cells were cultivated for 72 hours with the addition of 25 μM BPS (Sigma-Aldrich, USA), simulating the iron-deficient environment, or with 25 μM Fe-NTA (Sigma-Aldrich, USA) to ensure iron-rich conditions.

### Iron uptake

*N*. *fowleri* cells grown for 72 hours under iron-rich and iron-deficient conditions were washed with phosphate buffered saline (PBS) (1000 g for 15 min) and transferred to measuring buffer (50 mM glucose; 20 mM HEPES; pH 7.2). Cells were counted using a Guava easyCyte 8HT flow cytometer (Merck, Germany), and 2.5×10^5^ cells were equally split onto a 24-well plate. To assess iron uptake, cells were supplemented with 2 μM ^55^Fe-citrate, 2 μM ^55^Fe-citrate with 1 mM ascorbate, or 6.3 μM ^55^Fe-transferrin. Samples were incubated at 37°C for 1 hour, and then EDTA was added to a final concentration of 1 mM to chelate extracellular iron. Cells were washed three times by measuring buffer, and the protein concentration was assessed using a BCA kit (Sigma-Aldrich, USA). Samples were diluted to equal concentrations and separated using the Novex Native PAGE Bis–Tris Gel system (4–16%; Invitrogen, USA). The gel was vacuum-dried for 2 hours and autoradiographed using a tritium storage phosphor screen. The experiment was performed in three independent replicates. If applicable, densitometry was used to compare signal strength, using Fiji distribution of ImageJ [51].

### Ferric reductase activity

To assess the activity of *N*. *fowleri* ferric reductase under different iron conditions, a ferrozine assay was used to compare the formation of ferrous iron, as described previously [52]. Cells were grown under iron-rich and iron-deficient environments for 72 hours. Samples containing no cells and samples without Fe-EDTA were used as controls. The cells were washed twice and resuspended in glucose buffer (50 mM glucose, 0.5 mM MgCl_2_, 0.3 mM CaCl_2_, 5.1 μM KH_2_PO_4_, 3 μM Na_2_HPO_4_, pH 7.4). The total amount of proteins was assessed using a BCA kit, and samples were diluted to equal concentrations. All further work was performed with minimum light exposure. Ferrozine was added to a total concentration of 1.3 mM and Fe-EDTA to a concentration of 0.5 mM. Samples were incubated at 37°C for three hours, pelleted (1000 g for 10 min) and the supernatant was used to determine the formation of the colored Fe(II)-ferrozine complex, accompanied by a change in absorbance at 562 nm using 1 cm cuvettes and a UV-2600 UV-VIS spectrophotometer (Shimadzu, Japan). The experiment was performed in three independent replicates.

### Hemin utilization

To assess the ability of *N*. *fowleri* to utilize hemin, 2×10^4^ cells were cultivated in each well of a 24-well plate in fresh growth medium. Cultures were supplemented with 50 μM BPS, 50 μM hemin, or 50 μM hemin and 50 μM BPS. Cells without chelator or hemin were used as a control. The cell concentration was measured every 24 hours for four days using a Guava easyCyte 8HT flow cytometer. The experiment was performed in four independent replicates.

### Comparative proteomic analysis

*N*. *fowleri* cells were cultivated in 75 cm^2^ aerobic cultivation flasks under iron-rich and iron-deficient environments for 72 h. For whole-cell proteomic analysis, cells were washed three times in PBS (1000 g, 15 min, 4°C) and pelleted. The experiment was performed in three independent replicates.

In addition, a membrane-enriched fraction was prepared. Approximately 1.5×10^7^ cells were harvested (1000 g, 15 min, 4°C), washed in 15 ml of sucrose-MOPS buffer (250 mM sucrose, 20 mM 3-morpholinopropanesulfonic acid, pH 7.4, supplemented with complete EDTA-free protease inhibitors, Roche, Switzerland) and resuspended in 4 ml of sucrose-MOPS buffer. The suspension was sonicated using a Q125 sonicator (Qsonica, USA) (45% amplitude, total time of 4 min using 1 s pulse and 1 s pause). The resulting suspension was centrifuged (5000 g, 5 min) to spin down the unlysed cells. The obtained supernatant was further centrifuged (200 000 g, 20 min). The supernatant was discarded, and the membrane-enriched pellet was washed with distilled water. The experiment was performed in three independent replicates.

Label-free proteomic analysis of the samples was assessed using the method described in *Mach et al*. *2018* [34], utilizing liquid chromatography coupled with mass spectrometry. The resulting MS/MS spectra were compared with the *Naegleria fowleri* database, downloaded from amoebaDB [53] on 25/7/2017. The set thresholds to filter proteins were Q-value = 0, unique peptides detected >2, and the protein had to be identified at least twice in one condition from the six runs. For proteins identified only in one of the conditions, intensity of 23 was selected as a lowest value to be included, on basis of previous imputation experience. To distinguish significantly downregulated or upregulated proteins in the iron-deficient conditions in S1 and S2 Tables, the threshold of fold change >2.3 and <-2.3 was chosen, based on previous experience and published results on comparative proteomics in *N*. *gruberi* and *Ostreococcus tauri* [34,54].

The annotations and identifications of the selected proteins discussed in this study were confirmed using the tool HHPRED [55]. In addition, alignments were constructed using the chosen proteins and their homologues from different organisms: NF0014630 identified as mitochondrial carnitine/acylcarnitine transferase [56], NF0001420 identified as mitochondrial phosphate carrier [57], NF0060430 identified as IscU [58,59] and NF0079420 identified as mitoferrin [60,61]. Alignments with the indicated conserved sequences are shown in S5 Fig and were constructed using Geneious version 11.1.5 software with the muscle alignment tool.

### Western blot

To analyze the expression of *N*. *fowleri* hemerythrin in different iron conditions, cells cultivated in iron-rich and iron-deficient conditions for 72 hours were pelleted, washed by PBS and protein concentration was assessed using BCA kit. Equal amounts were boiled in SDS sample buffer (Merck Millipore, USA) for 5 min and samples were separated using sodium dodecyl sulphate–polyacrylamide gel electrophoresis as first described in [62]. Proteins were transferred to nitrocellulose membrane by western blotting using semi-dry method, for 75 minutes under 1.5 current of mA/cm^2^. The proteins were visualized by Ponceau S stain (0.5% Ponceau S, Merck Millipore, USA, 1% acetic acid), confirming a unified loading of the two samples.

The membrane was blocked for one hour, using 5% dried fat-free milk and 0.05% Tween 20 (Sigma-Aldrich, USA) in PBS. Afterwards, the blot was transferred to fresh blocking solution with primary antibody in ratio 500:1 for one hour. The polyclonal antibody was made in-house, against the whole protein (13.5 kDa) in rat with no adjuvants and was previously used in [34]. After washing with fresh blocking solution, secondary anti-rat antibody conjugated with horse-radish peroxidase (Sigma-Aldrich, USA) was added in 1:2000 ratio for one hour. After washing with fresh blocking buffer and PBS, the antibody was visualized using Chemiluminescent Peroxidase Substrate-1 (Sigma-Aldrich, USA) according to manufacturer protocol, on Amersham Imager 600 (GE Life Sciences, USA).

### Comparative transcriptomic analysis

To obtain the transcriptome data of *N*. *fowleri*, five independent replicates of approximately 1×10^6^ cells each were grown under iron-rich and iron-deficient conditions for 72 hours. A High Pure RNA Isolation Kit (Roche, Switzerland) was used to isolate cell RNA, and an Illumina-compatible library was prepared using QuantSeq 3′ mRNA-Seq Library Prep Kit FWD for Illumina (Lexogen, Austria). The RNA concentration was determined using a Quantus fluorometer (Promega, USA), and the quality of RNA was measured on a 2100 Bioanalyzer Instrument (Agilent technologies, USA). Equimolar samples were pooled to 10 pM and sequenced with MiSeq Reagent Kit v3 (Illumina, USA) using 150-cycles on the MiSeq platform. The obtained results were filtered using the *p*-value of >0.05 followed by analysis on the BlueBee platform with the method DESeq [63].

### Amino acid quantification assay

Approximately 3×10^6^ cells cultivated under iron-rich and iron-deficient environments were harvested by centrifugation (1000 g, 15 min, 4°C), washed with PBS supplemented with cOmplete EDTA-free protease inhibitor, and the total concentration of proteins was measured. Cells were transferred to 1 ml of buffer solution (20 mM Tris, 1 mM MgCl_2_, pH 8, cOmplete EDTA-free protease inhibitor) and sonicated with Sonopuls mini20 (Bandelin, Germany) (90% amplitude, 4°C, total time 120 s, 1 s pulse and 1 s pause). The resulting suspension was mixed at a ratio of 1:4 with ice-cold acetonitrile and maintained overnight at -20°C. Samples were centrifuged (16000 g, 20 min, 4°C) and filtered using Ultrafree Centrifugal Filter Units (Merck Millipore, USA). The experiment was performed in three independent replicates.

Samples were analyzed using liquid chromatography on a Dionex Ultimate 3000 HPLC system with on-line mass spectrometry detection (Thermo Scientific, USA). The separation was achieved using a HILIC column iHILIC-Fusion (150 x 2.1 mm, 1.8 μm particles, 100 Å pore size, HILICON, Sweden). The entire analysis flow rate was 0.3 ml/min, and the column was equilibrated with 100% of solution A (80% acetonitrile in water, 25 mM ammonium formate, pH 4.8) for 3 min. Amino acids were eluted by increasing the gradient of solution B (5% acetonitrile in water, 25 mM ammonium formate, pH 4.8), where 50% of solution B was reached in 7 min. After separation, the column was washed with 80% solution B for 3 min and then equilibrated with 100% solution A for 5 min.

Amino acids were detected by mass spectrometry using the triple quadrupole instrument TSQ Quantiva (Thermo Scientific, USA) in Selected Reaction Monitoring mode. Analytes were ionized using electrospray ionization on an H-ESI ion source and analyzed with positive charge mode with a spray voltage of 3500 V, ion transfer tube temperature of 325°C, and vaporizer temperature of 350°C. All transitions, collision energies, and RF voltages were optimized prior to analysis using appropriate amino acid standards. Each analyte was detected using at least two transitions. Cycle time was set to 1.8 s and both Q1 and Q3 resolutions were set to 0.7 s. To analyze the ion chromatograms and calculate peak areas, Skyline daily version 4.2.1.19004 [64] was used.

### Lactate production

To assess the difference in the intracellular production of lactate, 3×10^6^ cells cultivated under iron-rich or iron-deficient conditions were prepared for analysis in the same way as for quantifying the amino acid content. The experiment was performed in three independent replicates. After incubation with acetonitrile and filtration, the cell sample was dried and resuspended in 100 μl of anhydrous pyridine (Sigma-Aldrich, USA), and 25 μl of a silylation agent (N-tert-butyldimethylsilyl-N-methyl-trifluoroacetamide, Sigma-Aldrich, USA) was added. The sample was incubated at 70°C for 30 min. After incubation, 300 μl of hexane (Sigma-Aldrich, USA) and 10 μl of an internal standard (102 μg/ml 1-bromononane solution in hexane) were added. Selected compounds were analyzed as tert-butyl silyl derivatives.

Samples were analyzed using two-dimensional gas chromatography coupled with mass detection (GCxGC-MS; Pegasus 4D, Leco Corporation, USA) with ChromaTOF 4.5 software. Mass detection was equipped with an EI ion source and TOF analyzer with unite resolution. A combination of Rxi-5Sil (30 m x 0.25 mm, Restek, Australia) and BPX-50 (0.96 m x 0.1 mm, SGE, Australia) columns were used. The input temperature was set to 300°C, the injection volume was 1 μl in spitless mode, and constant helium flow of 1 ml/min, modulation time 3 s (hot pulse 1 s) and modulation temperature offset with respect to the secondary oven 15°C were used. The temperature program applied on the primary oven was 50°C (hold 1 min), which was increased by the rate of 10°C/min to a final temperature of 320°C (hold 3 min). The temperature offset applied on the secondary column was +5°C.

### Cell respiration

Five independent replicates of 3×10^6^ *N*. *fowleri* cells grown for 72 hours under iron-rich and iron-deficient conditions were washed twice and resuspended in 1 ml of glucose buffer, and the protein concentration was assessed using a BCA kit. Total cell respiration was measured as the decrease in oxygen concentration using an Oxygen meter model 782 (Strathkelvin instruments, UK) with Mitocell Mt 200 cuvette of total volume of 700 μl at 37°C. Measurements were carried out with 5×10^5^ cells for 5 min, after which potassium cyanide was added to a final concentration of 4 mM to block complex IV, and after 5 min, salicyl hydroxamic acid was added to a final concentration of 0.2 mM to completely block AOX. Values gained after the addition of potassium cyanide and salicyl hydroxamic acid were subtracted to acquire canonical respiratory chain and AOX activity, respectively.

### Hydrogenase and complex I activity levels

Approximately 1×10^6^ cells cultivated under iron-rich or iron-deficient conditions were washed and resuspended in 0.2 ml of saccharose-MOPS buffer (250 mM saccharose, 10 mM 3-morpholinopropanesulfonic acid, pH 7.2). To assess the hydrogenase activity, we used a protocol previously described [65]. Briefly, the reaction was initiated by the addition of cells lysed with 0.02% Triton X-100 to 2 ml of measuring buffer (0.1 M Tris; 50 mM KCl buffer, pH 7.4; 1 μM methylviologen and 0.5% β-mercaptoethanol, saturated with hydrogen gas). Activity level was assessed on the basis of the change in 600 nm absorbance using 1-cm quartz cuvettes on a Shimadzu UV-2600 UV-VIS spectrophotometer with UVProbe software (Shimadzu, Japan). To assess the complex I activity level, we used a protocol previously described [66]. Briefly, 1% digitonin-treated cells were added to 2 ml of measuring buffer (0.1 M KPi buffer, pH 7.5, and 0.2 mM NADH), and the reaction was initiated by the addition of 50 μM oxidized coenzyme Q2 (Sigma-Aldrich, USA) suspended in ethanol. Activity level was assessed on the basis of the change in 340 nm absorbance using 1-cm quartz cuvettes on the same spectrophotometer as used to assess hydrogenase activity. As a control, 0.2 mM rotenone was added after the measurement as a specific inhibitor of complex I to confirm that the background activity was negligible. Both experiments were performed in four independent replicates.

### Chelators

The growth dependence of *N*. *fowleri* on iron availability was defined using the iron chelators BPS (Sigma-Aldrich, USA), DIP (Sigma-Aldrich, USA) and DFO (Sigma-Aldrich, USA). 5000 *N*. *fowleri* cells per ml were cultivated in 24-well plates in a total volume of 1 ml in a humid chamber. Each chelator and control were tested in four independent replicates (final concentrations of 100, 50, 25, 12.5, 6.3, 3.1, 1.6 and 0.8 μM). Cells were cultivated for 48 hours, the plates were placed on ice for 10 min, and the medium was gently pipetted to detach the cells. The number of cells in each sample was counted using a Guava easyCyte 8HT flow cytometer. The value of half-maximal inhibitory concentration (IC_50_) was calculated using the online calculator on the AAT Bioquest webpage [67]. Graphs were created using GraphPad Prism 6 (GraphPad software, USA).

Growth curves of the organism under the influence of different iron chelators were constructed by inoculating 5000 cells/ml *N*. *fowleri* trophozoites into 10 ml of cultivation medium with the appropriate compound (25 μM Fe-NTA; 45 μM DIP; 25 μM BPS or 10 μM DFO) in four independent replicates. Counting the number of the cells in culture at 24, 48 and 72 hours was performed by flow cytometry using a Guava easyCyte 8HT flow cytometer.

### *N*. *fowleri* bacterial phagocytosis

Approximately 3×10^6^ cells cultivated under iron-rich and iron-deficient conditions were washed in cultivation flasks by replacing the growth medium with 10 ml of PBS warmed to 37°C and resuspending in 7 ml of 37°C PBS. To assess the ability of the cells to phagocytose, 150 μl of pHrodo Green *E*. *coli* BioParticles Conjugate for Phagocytosis (Thermo Fisher Scientific, USA) was added, and the cells were incubated for 3 hours at 37°C. Following incubation, the cells were washed with PBS, detached on ice for 15 min, and the fluorescence caused by the phagocytosed particles was consecutively analyzed using a Guava easyCyte 8HT flow cytometer using a 488 nm laser and a Green-B 525/30 nm detector. A negative control (without the addition of BioParticles) was used to determine an appropriate threshold for *N*. *fowleri* cells. BioParticles resuspended in PBS were measured in the same way to determine the background noise and gave a negligible signal. The effect of different iron availability on the efficiency of phagocytosis of *N*. *fowleri* was established as the percentage of cells in culture that had increased fluorescence. The experiment was performed with nine independent replicates.

To visualize the ability of *N*. *fowleri* to phagocytize, live amoebae incubated with fluorescent *E*. *coli* were imaged with a Leica TCS SP8 WLL SMD-FLIM microscope (Leica, Germany) equipped with an HC PL APO CS2 63x/1.20 water objective with 509 nm excitation, 526 nm-655 nm excitation was detected with a HyD SMD detector, and a PMT detector was used for brightfield imaging. Images were processed using LAS X 3.5.1.18803 (Leica, Germany).

To test the effect of attenuated strain of bacteria on the propagation of iron-starved *N*. *fowleri* in culture, we preincubated amoebae under iron-rich and iron-deficient conditions for 72 hours and inoculated approximately 1000 cells into 96-well plates to a total volume of 200 μl under select conditions with or without the equivalent of 1×10^6^ *Enterobacter aerogenes* that had been attenuated. Preincubated iron-rich cells were inoculated into iron-rich medium as a control, and simultaneously, preincubated iron-deficient cells were inoculated into either iron-deficient or iron-rich medium. The cells were cultivated in a humid chamber at 37°C for 48 hours and then counted using a Guava easyCyte 8HT flow cytometer. The experiment was performed with six independent replicates.

## Supporting information

S1 Fig **(A) Loading control corresponding to Fig 1A, ferrous and ferric iron uptake by *N*. *fowleri* precultivated under iron-rich and iron-deficient conditions.** Coomassie brilliant blue loading stain showed that equal protein concentrations in the samples were subjected to native electrophoresis gels. The proteins were determined from whole cell extracts of *N*. *fowleri* previously cultivated for 72 hours under iron-deficient conditions (25 μM BPS) or iron-rich conditions (25 μM Fe-NTA) and further incubated with ^55^Fe(II) (ferrous ascorbate) and ^55^Fe(III) (ferric citrate). **(B) Lack of** ^**55**^**Fe-transferrin uptake in *N*. *fowleri*, cultivated under iron-rich and iron-deficient conditions.** The uptake of transferrin-bound iron was assessed by incubation of *N*. *fowleri* with ^55^Fe-transferrin. Tf, pure ^55^Fe-transferrin; Fe, *N*. *fowleri* cultivated under iron-rich conditions for 72 hours, consecutively incubated with ^55^Fe-transferrin for 1 hour; BPS, *N*. *fowleri* cultivated under iron-deficient conditions for 72 hours, consecutively incubated with ^55^Fe-transferrin for 1 hour; Ctr, iron uptake control of *N*. *fowleri* culture cultivated in iron deficiency incubated with ^55^Fe(III)-citrate for 1 hour. The utilization of iron was analyzed by blue native electrophoresis as described in the Methods section. Equal protein concentrations were loaded, as shown on the Coomassie brilliant blue loading stain. Gel is a representative from three independent replicates. **(C) Mechanism of ferric iron uptake involves the reductive step.** *N*. *fowleri* culture was incubated for 1 hour with ^55^Fe(III)-citrate with and without the addition of 0.2 mM BPS. Incorporation of ^55^Fe(III)-citrate to cellular proteins was higher in the sample without the presence of BPS, indicating that a reductive iron uptake mechanism takes place. Several distinct signals on the lower part of +BPS probably correspond to residues of BPS complexed with ferrous iron radionuclides. The utilization of iron was analyzed by blue native electrophoresis as described in the Methods section. -BPS, cell sample without addition of BPS chelator; +BPS, cell sample with the addition of BPS chelator. Equal protein concentrations were loaded, as shown on the Coomassie brilliant blue loading stain. Gel is a representative from three independent replicates. **(D) Loading control corresponding to Fig 2A, *N*. *fowleri* cell lysate for Western blot analysis of hemerythrin expression.** Ponceau S loading stain shows equal protein concentrations of loaded samples of *N*. *fowleri* cultivated under iron-deficient (BPS) and iron-rich (Fe) conditions.
(TIF)

S2 Fig **(A) A representative growth curve of *N*. *fowleri* treated with different chelators.** The chelators hindered the propagation of the cells in culture. The graphs show the cytostatic effect of chosen concentrations of the iron chelators compared with the effect of iron-rich cultivation conditions. Fe, cells cultivated under iron-rich conditions (25 μM Fe-NTA); DIP, cells cultivated in 45 μM DIP; BPS, cells cultivated in 25 μM BPS; and DFO, cells cultivated in 10 μM DFO. Data are presented as the means ± SD from four independent replicates. **(B) *N*. *fowleri* growth in different concentrations of chelators after 48 hours.** The shown graphs were used to calculate the IC_50_ values for different chelators. The graph was created using GraphPad Prism 6 (GraphPad software, USA). Data are presented as the means ± SD from four independent replicates.
(TIF)

S3 Fig *N. fowleri* cell phagocytosis. Representative dot plots of flow cytometry results of *N*. *fowleri* phagocyting bacteria using a pHrodo green *E*. *coli* BioParticles conjugate to measure phagocytosis (Thermo Fisher Scientific, USA) in nine independent replicates. *N*. *fowleri*, control culture with no added bacteria; Bacteria, control for the bacteria cells; *N*. *fowleri* Fe, *N*. *fowleri* under iron-rich conditions with added bacteria; *N*. *fowleri* BPS, *N*. *fowleri* under iron-deficient conditions with added bacteria.
(TIF)

S4 Fig Phagocytosis of bacteria is not an iron acquisition strategy of *N. fowleri*. Effect of adding the attenuated bacteria *Enterobacter aerogenes* on the propagation of *N*. *fowleri* under different iron conditions. After 48 hours, amoebae growth was not changed when bacteria were added under any condition (iron-rich cells, iron-deficient cells or cells preincubated under iron-deficient conditions and subsequently transferred into an iron-rich environment all had p-values >0.05). The propagation of the amoebae in the iron-deficient culture was significantly lower than that in the iron-rich culture (23% with bacteria and 26% without bacteria, p-values <0.01 for both), confirming the effect of iron deficiency on amoeba culture propagation. Furthermore, cultures preincubated under iron-deficient conditions and subsequently transferred into iron-rich environments had the same propagation as those under the iron-rich culture conditions (p-values >0.05 with and without bacteria). Data are presented as the means ± SD from six independent replicates.
(TIF)

S5 Fig Alignments of *N. fowleri* proteins with homologues from other organisms. (A) Alignment of *N*. *fowleri* NF0060430 with the IscU proteins from *Homo sapiens*, *Saccharomyces cerevisiae* and *T*. *brucei*. Red arrows point to the conserved cysteine required for iron-sulfur cluster assembly, based on a previous study [58]. The red rectangle denotes the conserved LPPVK motif of the IscU proteins [59]. (B) Alignment of *N*. *fowleri* NF0079420 with the mitoferrin proteins of *Trypanosoma brucei*, *Leishmania mexicana*, *Saccharomyces cerevisiae* and *Homo sapiens*. Red arrows point to the sequence motif Px(D/E)xx(K/R)x(K/R), and yellow circles mark residues in contact with substrate, according to a previous study [60]. Conserved histidine residues responsible for iron transport are marked with blue stars [61]. (C) Alignment of *N*. *fowleri* NF0001420 with the mitochondrial phosphate carriers of *Saccharomyces cerevisiae*, *Homo sapiens* and *Arabidopsis thaliana*. Red arrows point to residues important for the phosphate transport activity, according to a previous study [57]. (D) Alignment of *N*. *fowleri* NF0014630 with mitochondrial carnitine/acylcarnitine transferases of *Saccharomyces cerevisiae*, *Arabidopsis thaliana*, *Homo sapiens* and *Caenorhabditis elegans*. The red rectangle denotes the signature motifs Px(D/E)xx(R/K)x(R/K), and the arrows point to conserved residues, according to a previous study [56].
(TIF)

S1 Table Comparison of whole-cell proteomes of *N. fowleri* in iron-rich and iron-deficient environments. List of whole-cell proteomes of *N*. *fowleri* compared in iron-rich and iron-deficient conditions sorted into four sheets: raw data, all detected proteins, significantly upregulated proteins and significantly downregulated proteins under iron-deficient conditions. With the exception of raw data, the tables are simplified to show only fold change values. Proteins with > 2.3 (denoting upregulated under iron-deficient conditions) or < -2.3-fold change (denoting downregulated under iron-deficient conditions) are regarded as significantly regulated. Proteins were annotated from amoebaDB [53] on 25/7/2017, and manual annotation was performed for selected proteins, as described in the Methods section. Probable iron-containing proteins of the significantly downregulated and upregulated proteins are highlighted in yellow. Experiment was performed with three independent replicates.
(XLSX)

S2 Table Comparison of membrane-enriched proteomes of *N. fowleri* in iron-rich and iron-deficient environments. List of membrane-enriched proteomes of *N*. *fowleri* compared in iron-rich and iron-deficient conditions sorted into four sheets: raw data, all detected proteins, significantly upregulated proteins and significantly downregulated proteins under iron-deficient conditions. With the exception of raw data, the tables are simplified to show only fold change values. Proteins with >2.3 (denoting upregulated under iron-deficient conditions) or < -2.3-fold change (denoting downregulated under iron-deficient conditions) are regarded as significantly regulated. Proteins were annotated from amoebaDB [53] on 25/7/2017, and manual annotation was performed for selected proteins, as described in the Methods section. Experiment was performed with three independent replicates.
(XLSX)

S3 Table Comparison of transcriptomes of *N. fowleri* in iron-rich and iron-deficient environments. List of genes significantly downregulated and upregulated under iron-deficient conditions. Raw data are included, and manual annotation was performed for selected proteins, as described in the Methods section. Experiment was performed with five independent replicates.
(XLSX)

S4 Table Raw data. (XLSX)

S1 Video The video shows a demonstration of *N. fowleri* phagocytizing bacteria. In the video, a single *N*. *fowleri* amoeba phagocytoses several fluorescently modified pHrodo green *E*. *coli* BioParticles (bright blue). In the lysosomes, they fluoresced due to the decreased pH value. In the background, several non-fluorescing bacteria were observed (dim blue). Images were acquired with a Leica TCS SP8 WLL SMD-FLIM microscope (Leica, Germany) equipped with an HC PL APO CS2 63×/1.20 water objective with 509 nm excitation, 526 nm-655 nm excitation was detected with a HyD SMD detector, and a PMT detector was used for brightfield imaging. Images were processed using LAS X 3.5.1.18803 (Leica, Germany). Video was created and edited using the Fiji distribution package of ImageJ software [51].
(MP4)
