# Supplementary figures and images for: Adaptive iron utilization compensates for the lack of an inducible uptake system in Naegleria fowleri and represents a potential target for therapeutic intervention
Source: PLoS Negl Trop Dis. 2020 Jun 18;14(6):e0007759. doi: 10.1371/journal.pntd.0007759 (PMC7326272; doi:10.1371/journal.pntd.0007759)

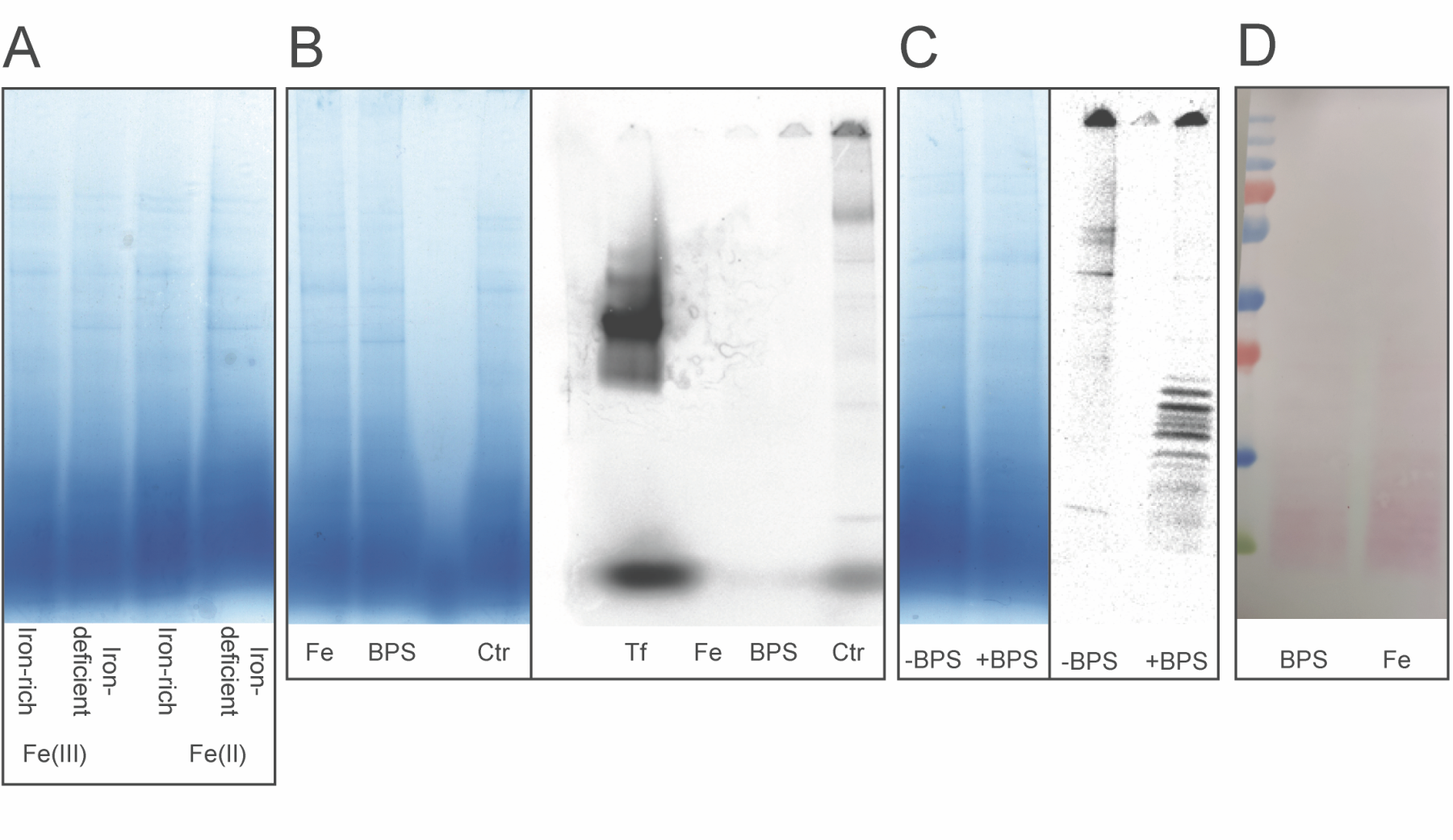

Supplement: S1 Fig — (A) Loading control corresponding to Fig 1A, ferrous and ferric iron uptake by N. fowleri precultivated under iron-rich and iron-deficient conditions. Coomassie brilliant blue loading stain showed that equal protein concentrations in the samples were subjected to native electrophoresis gels. The proteins were determined from whole cell extracts of N. fowleri previously cultivated for 72 hours under iron-deficient conditions (25 μM BPS) or iron-rich conditions (25 μM Fe-NTA) and further incubated with 55Fe(II) (ferrous ascorbate) and 55Fe(III) (ferric citrate). (B) Lack of 55Fe-transferrin uptake in N. fowleri, cultivated under iron-rich and iron-deficient conditions. The uptake of transferrin-bound iron was assessed by incubation of N. fowleri with 55Fe-transferrin. Tf, pure 55Fe-transferrin; Fe, N. fowleri cultivated under iron-rich conditions for 72 hours, consecutively incubated with 55Fe-transferrin for 1 hour; BPS, N. fowleri cultivated under iron-deficient conditions for 72 hours, consecutively incubated with 55Fe-transferrin for 1 hour; Ctr, iron uptake control of N. fowleri culture cultivated in iron deficiency incubated with 55Fe(III)-citrate for 1 hour. The utilization of iron was analyzed by blue native electrophoresis as described in the Methods section. Equal protein concentrations were loaded, as shown on the Coomassie brilliant blue loading stain. Gel is a representative from three independent replicates. (C) Mechanism of ferric iron uptake involves the reductive step. N. fowleri culture was incubated for 1 hour with 55Fe(III)-citrate with and without the addition of 0.2 mM BPS. Incorporation of 55Fe(III)-citrate to cellular proteins was higher in the sample without the presence of BPS, indicating that a reductive iron uptake mechanism takes place. Several distinct signals on the lower part of +BPS probably correspond to residues of BPS complexed with ferrous iron radionuclides. The utilization of iron was analyzed by blue native electrophoresis as de [file pntd.0007759.s001.tif]

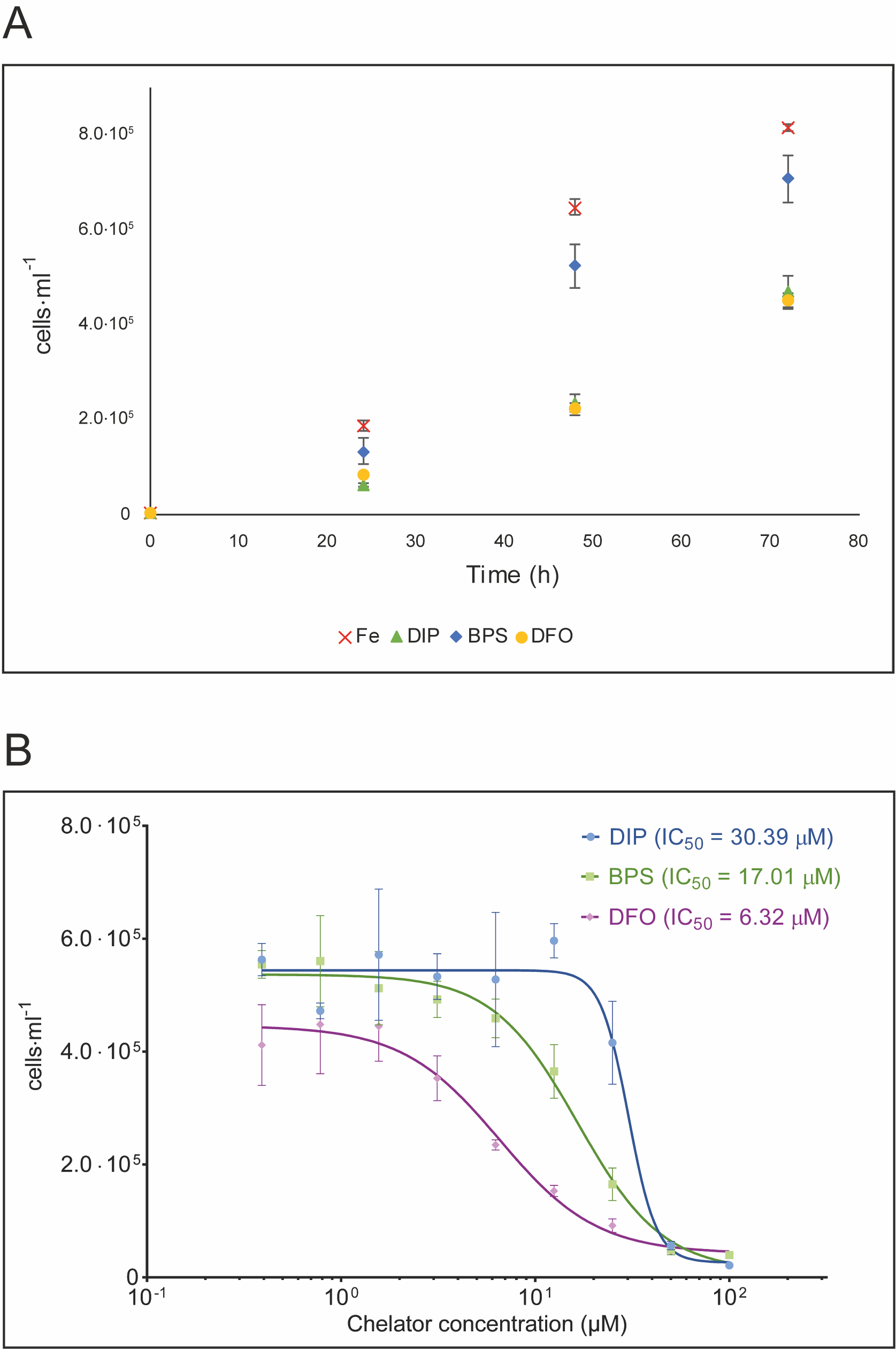

Supplement: S2 Fig — (A) A representative growth curve of N. fowleri treated with different chelators. The chelators hindered the propagation of the cells in culture. The graphs show the cytostatic effect of chosen concentrations of the iron chelators compared with the effect of iron-rich cultivation conditions. Fe, cells cultivated under iron-rich conditions (25 μM Fe-NTA); DIP, cells cultivated in 45 μM DIP; BPS, cells cultivated in 25 μM BPS; and DFO, cells cultivated in 10 μM DFO. Data are presented as the means ± SD from four independent replicates. (B) N. fowleri growth in different concentrations of chelators after 48 hours. The shown graphs were used to calculate the IC50 values for different chelators. The graph was created using GraphPad Prism 6 (GraphPad software, USA). Data are presented as the means ± SD from four independent replicates. (TIF) [file pntd.0007759.s002.tif]

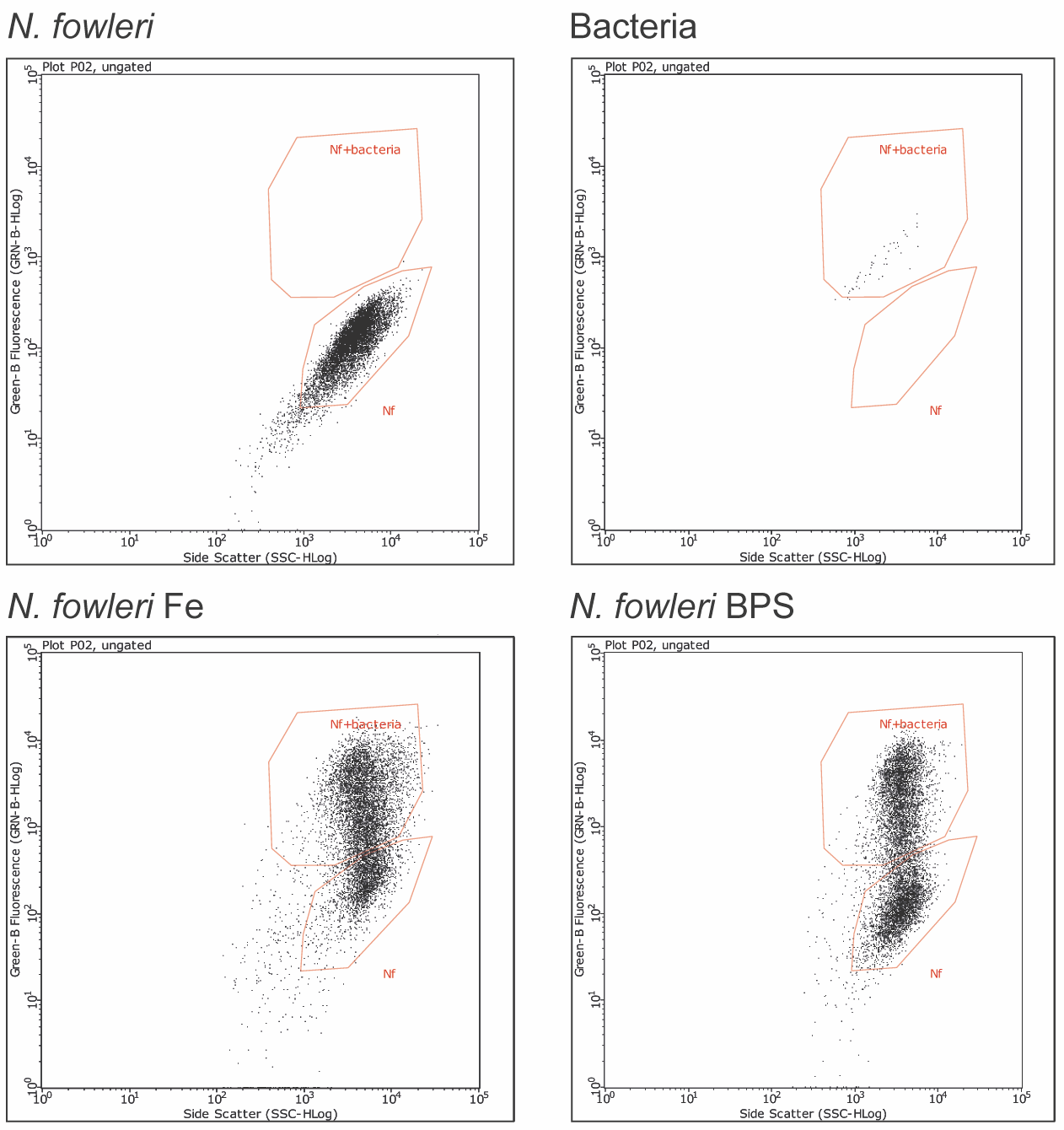

Supplement: S3 Fig — Representative dot plots of flow cytometry results of N. fowleri phagocyting bacteria using a pHrodo green E. coli BioParticles conjugate to measure phagocytosis (Thermo Fisher Scientific, USA) in nine independent replicates. N. fowleri, control culture with no added bacteria; Bacteria, control for the bacteria cells; N. fowleri Fe, N. fowleri under iron-rich conditions with added bacteria; N. fowleri BPS, N. fowleri under iron-deficient conditions with added bacteria. (TIF) [file pntd.0007759.s003.tif]

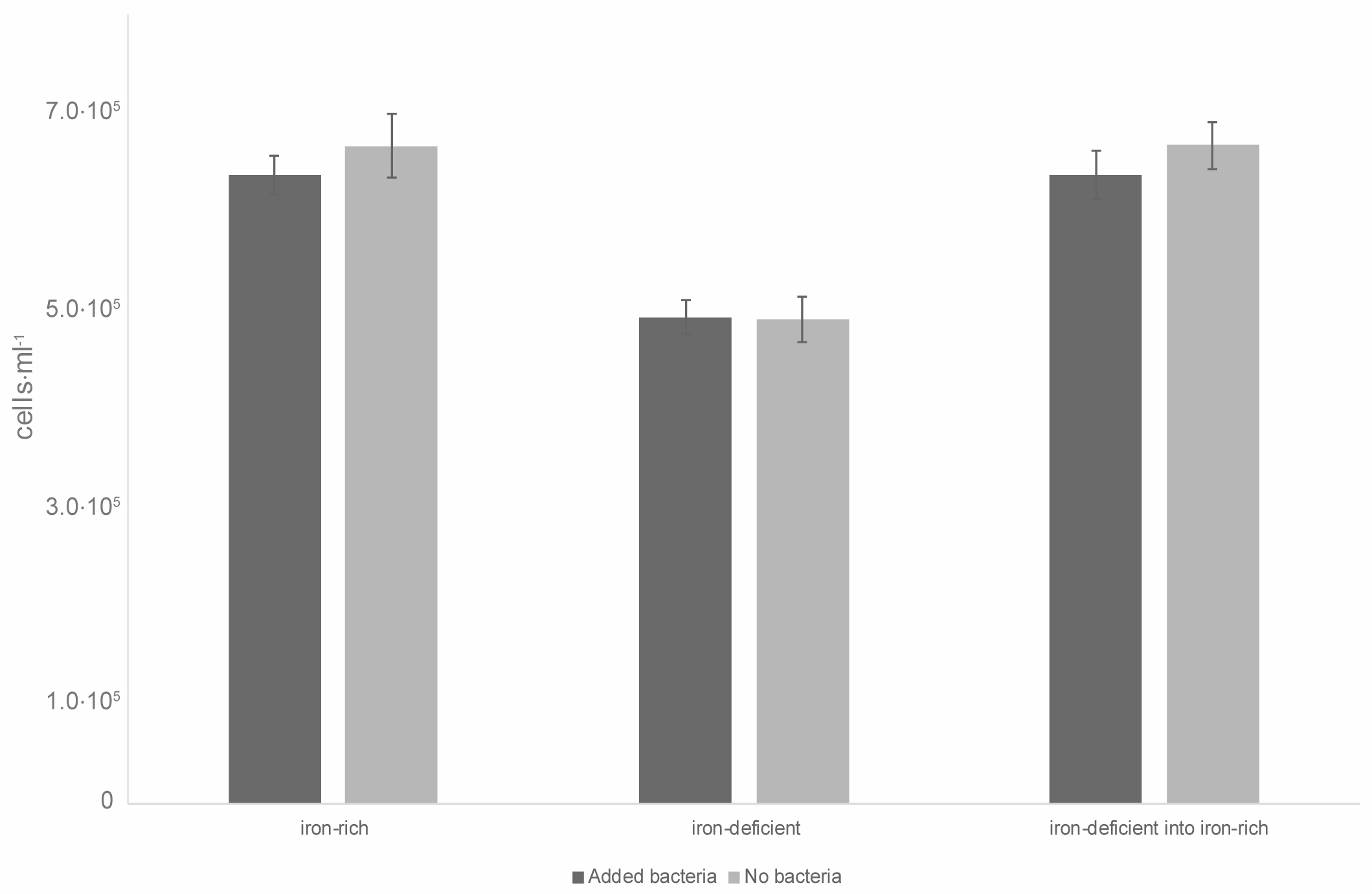

Supplement: S4 Fig — Effect of adding the attenuated bacteria Enterobacter aerogenes on the propagation of N. fowleri under different iron conditions. After 48 hours, amoebae growth was not changed when bacteria were added under any condition (iron-rich cells, iron-deficient cells or cells preincubated under iron-deficient conditions and subsequently transferred into an iron-rich environment all had p-values >0.05). The propagation of the amoebae in the iron-deficient culture was significantly lower than that in the iron-rich culture (23% with bacteria and 26% without bacteria, p-values <0.01 for both), confirming the effect of iron deficiency on amoeba culture propagation. Furthermore, cultures preincubated under iron-deficient conditions and subsequently transferred into iron-rich environments had the same propagation as those under the iron-rich culture conditions (p-values >0.05 with and without bacteria). Data are presented as the means ± SD from six independent replicates. (TIF) [file pntd.0007759.s004.tif]

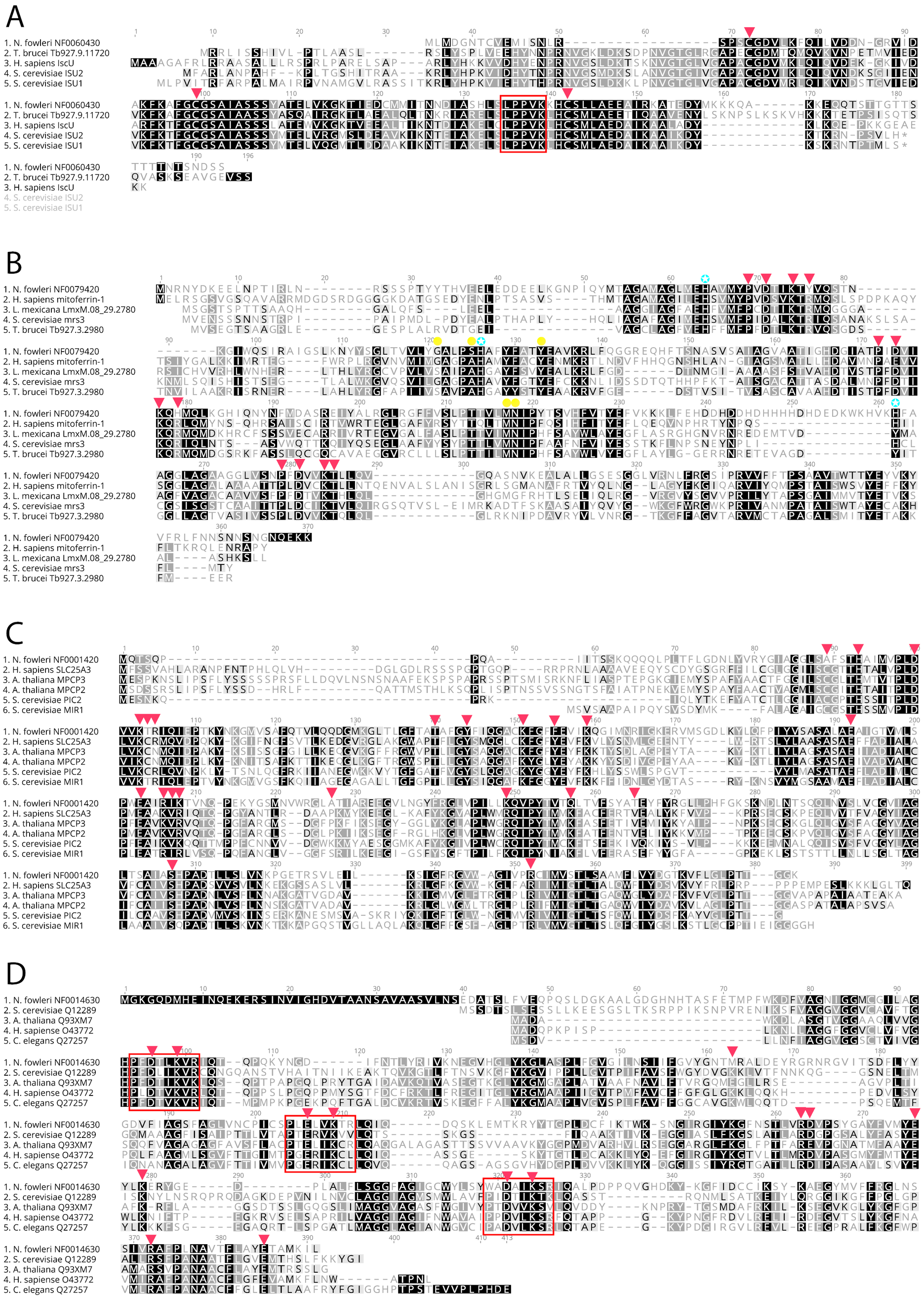

Supplement: S5 Fig — (A) Alignment of N. fowleri NF0060430 with the IscU proteins from Homo sapiens, Saccharomyces cerevisiae and T. brucei. Red arrows point to the conserved cysteine required for iron-sulfur cluster assembly, based on a previous study [58]. The red rectangle denotes the conserved LPPVK motif of the IscU proteins [59]. (B) Alignment of N. fowleri NF0079420 with the mitoferrin proteins of Trypanosoma brucei, Leishmania mexicana, Saccharomyces cerevisiae and Homo sapiens. Red arrows point to the sequence motif Px(D/E)xx(K/R)x(K/R), and yellow circles mark residues in contact with substrate, according to a previous study [60]. Conserved histidine residues responsible for iron transport are marked with blue stars [61]. (C) Alignment of N. fowleri NF0001420 with the mitochondrial phosphate carriers of Saccharomyces cerevisiae, Homo sapiens and Arabidopsis thaliana. Red arrows point to residues important for the phosphate transport activity, according to a previous study [57]. (D) Alignment of N. fowleri NF0014630 with mitochondrial carnitine/acylcarnitine transferases of Saccharomyces cerevisiae, Arabidopsis thaliana, Homo sapiens and Caenorhabditis elegans. The red rectangle denotes the signature motifs Px(D/E)xx(R/K)x(R/K), and the arrows point to conserved residues, according to a previous study [56]. (TIF) [file pntd.0007759.s005.tif]
